# Computed tomography and magnetic resonance imaging radiomics for predicting non-complete response after chemoradiotherapy in unresectable locally advanced head and neck squamous cell carcinoma

**DOI:** 10.1016/j.phro.2026.101074

**Published:** 2026-09-05

**Authors:** Krittee Chesdachai, Kullathorn Thephamongkhol, Anucha Chaichana, Jiraporn Setakornnukul

**Affiliations:** a Division of Radiation Oncology, Department of Radiology, Faculty of Medicine Siriraj Hospital, Mahidol University, Bangkok, Thailand; b Department of Radiological Technology, Faculty of Medical Technology, Mahidol University, Bangkok, Thailand

**Keywords:** Head and neck squamous cell carcinoma, Radiomics, Magnetic resonance imaging, Definitive chemoradiotherapy, Treatment response prediction

## Abstract

**Background and purpose:**

Non-complete response after chemoradiotherapy in unresectable locally advanced head and neck squamous cell carcinoma (LA-HNSCC) has been associated with poor prognosis. We compared clinical, computed tomography (CT), and magnetic resonance imaging (MRI) radiomics models for predict non-complete response of the primary tumor, and estimated each data type's marginal contribution across all configurations.

**Materials and methods:**

This retrospective study included 88 patients (67 complete and 21 non-complete responders) treated with definitive chemoradiotherapy. Radiomic features were extracted from pre-treatment contrast-enhanced CT and multi-parametric MRI. Models were optimized under nested leave-one-out cross-validation across 366 configurations spanning imbalance handling, feature selection, and classifier. Each data type's marginal contribution was estimated as the mean paired AUC difference versus a parsimonious clinical model. Early and late fusion were tested, and clinical utility was assessed by exploratory decision curve analysis.

**Results:**

In a best-configuration comparison, the MRI model achieved the highest discrimination (AUC 0.91, 95% CI 0.81–0.98) but did not significantly outperform the parsimonious clinical model (AUC 0.75, DeLong *p* = 0.058). Across all configurations, no single-modality or feature-level fusion exceeded the clinical baseline, each performing significantly below it (all *p* < 0.05). Only decision-level fusion combining clinical and imaging data was positive, reaching significance for clinical plus CT (mean ΔAUC +0.059, *p* = 0.031).

**Conclusions:**

A properly specified clinical model was a strong benchmark for radiomics. Single-best pipeline results can overstate radiomic value in small cohorts, and biascontrolled evaluation with external validation is essential before clinical use.

## Highlights

• Non-complete response to head and neck chemoradiotherapy is hard to predict early.
• Clinical, CT and MRI radiomics compared by marginal contribution across pipelines.
• A parsimonious clinical model with tumor volume was a strong predictive baseline.
• Decision-level fusion of clinical and CT data added value beyond the baseline.
• Single best-pipeline results overstate the added value of radiomics in small cohorts.

## Graphical abstract

**Figure:**
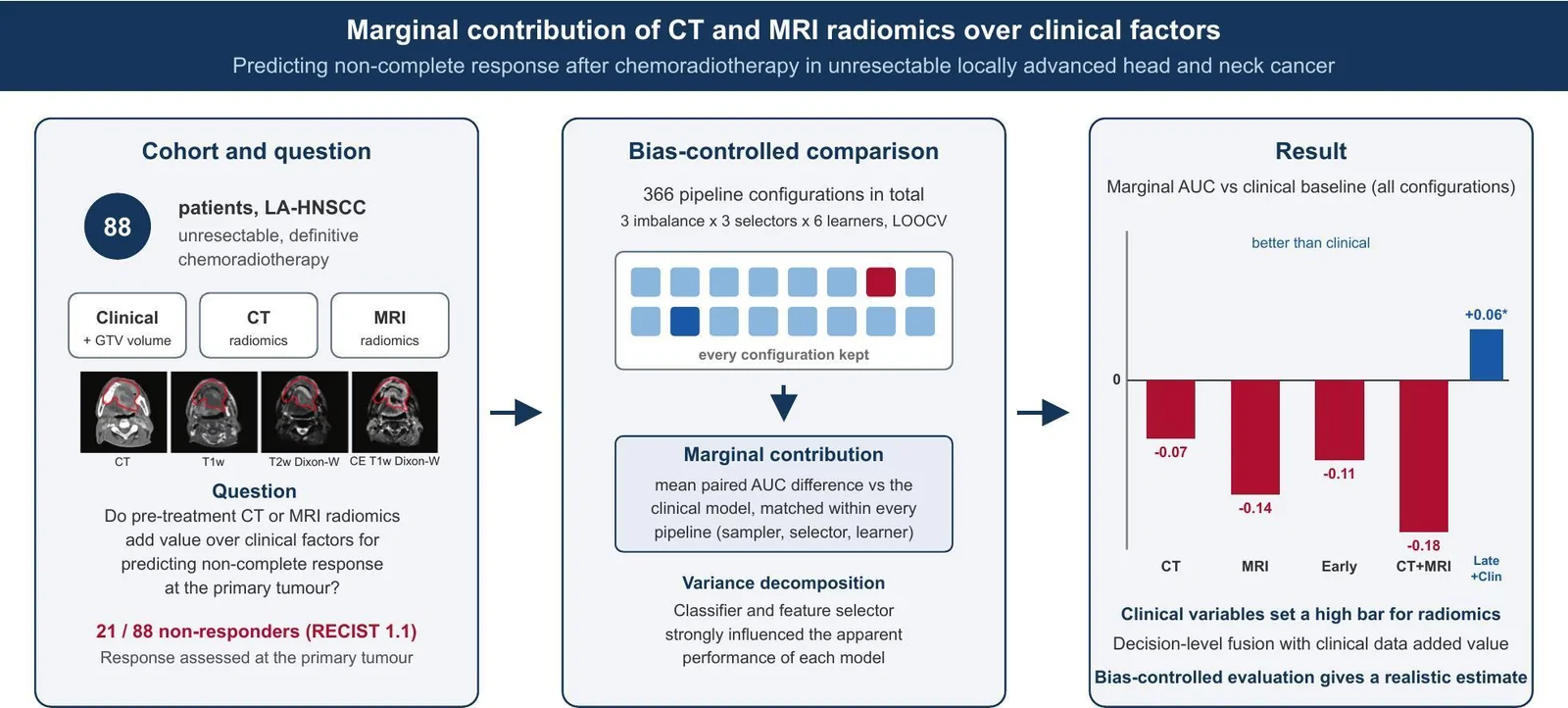

## 1 Introduction

Definitive concurrent chemoradiotherapy is the standard treatment for unresectable locally advanced head and neck squamous cell carcinoma (LA-HNSCC) [1], but locoregional control and overall survival remain suboptimal [2]. Most recurrences occur within the high-dose region, suggesting radioresistant subregions within the primary tumor [3]. Prognosis in LA-HNSCC has traditionally been based on clinical factors such as primary site and tumor stage, yet considerable variability in outcomes persists among patients with similar disease profiles, reflecting pronounced intra-tumoral heterogeneity [4], [5]. This heterogeneity likely reflects the coexistence of radiosensitive and radioresistant subregions within the gross tumor [6]. Achieving a clinical complete response (CR) after completion of chemoradiation is a key predictor of overall survival; conversely, a non-complete response (nCR) is associated with a substantially worse prognosis [7]. Early identification of patients at high risk of nCR could enable risk-adapted treatment strategies [8].

Radiomics converts medical images into high-dimensional quantitative descriptors that are not visible to the naked eye, and has shown promise for outcome prediction in oncology [9], [10]. MRI, with its superior soft-tissue contrast, is increasingly used in radiotherapy planning for head and neck cancer and is well-suited for radiomic analysis [11], [12]. Several studies have reported that radiomic signatures derived from CT, MRI, or PET can predict clinical outcomes in HNSCC, and MRI-based models have shown promising performance [5], [13], [14]. However, the relative value of MRI versus CT radiomics has not been evaluated in systematic head-to-head comparisons, and few studies have assessed clinical net benefit beyond discrimination metrics. Feature reproducibility and generalizability remain major challenges, as many radiomic features are sensitive to variations in acquisition parameters and preprocessing [14], [15].

Therefore, we aimed to develop and compare clinical, CT-based, and MRI-based radiomic machine-learning models to predict clinical non-complete response after definitive chemoradiotherapy in unresectable LA-HNSCC, using a reproducible methodology with rigorous feature selection and standardization. We further investigated whether incorporating multimodal data through early and late fusion strategies improved prediction and assessed clinical utility through decision curve analysis. This approach is intended to capture both multi-scale and structural dimensions of tumor heterogeneity, thereby supporting more individualized, risk-adapted management and potentially guiding future dose-painting or treatment-intensification strategies in patients at high risk of non-complete response.

## 2 Materials and methods

### 2.1 Patient cohort and endpoint

This study was approved by the Siriraj Institutional Review Board (CoA No. 750/2024; protocol No. MU-MOU 692/2567). The retrospective cohort comprised 88 patients with biopsy-proven stage III or IV squamous cell carcinoma of the head and neck (oral cavity, oropharynx, larynx, hypopharynx) without distant metastasis who received definitive concurrent chemoradiotherapy between August 2022 and May 2024. HPV testing was performed only in the oropharyngeal subsite. All patients had pre-treatment CT and MRI scans. Patients with prior cancer, prior head and neck irradiation, incomplete treatment, or prior surgery other than biopsy were excluded. Radiotherapy was delivered by volumetric modulated arc therapy to a median EQ.D2 of 70.7 Gy, with concurrent platinum-based chemotherapy (Supplementary Table S1).

Response at the primary tumor (the radiomic target) was assessed 3–6 months after treatment (median, 96 days; IQR, 84–122) by the Response Evaluation Criteria in Solid Tumors (RECIST 1.1) [16] on follow-up CT (70/88) or MRI (18/88), with clinical examination of the primary site. Modality choice reflected physician practice and scanner availability rather than suspicion of residual disease. Complete response was classified as CR, and partial response, stable disease, or progressive disease as nCR, the study endpoint and positive class for modeling.

### 2.2 Image acquisition and segmentation

Pre-treatment CT and MRI scans were performed using standardized clinical protocols (median simulation-to-treatment interval, 14 days; IQR, 14–15 days). Contrast-enhanced CT (CE-CT) used Siemens SOMATOM Confidence RT or Philips Big-bore scanners (120 kV, 2 mm slice thickness, 500 mm FOV, 512 × 512 matrix). Multi-parametric MRI used a Philips Ingenia 3 T scanner and included pre-contrast T1-weighted (T1w), T2-weighted Dixon with water-only reconstruction (T2w Dixon-W), and post-contrast T1-weighted Dixon (CE T1w Dixon-W). Contrast agents (iopromide, gadobutrol), doses, and acquisition parameters are in Supplementary Tables S1 and S2. The primary gross tumor volume (GTVp) was manually contoured on T2w Dixon-W by a radiation oncologist experienced in head and neck cancers, then propagated and adjusted on each remaining image using the Eclipse software (Supplementary Fig. S1).

### 2.3 Image preprocessing and feature extraction

Preprocessing comprised 3D recursive Gaussian denoising (sigma 0.5 mm), N4ITK bias-field correction [17] for MRI, and resampling of all volumes and masks to isotropic 1 × 1 × 1 mm^3^ voxels by B-spline interpolation. MRI intensities were *Z*-score normalized (scaled by 100) using the mean and standard deviation of the entire image. CT images remained in Hounsfield Units (HU) with a voxel array shift of 1024.

Radiomic features were extracted from the Primary Tumor ROI (GTVp) using PyRadiomics (v3.1.1) [18]. Three image types were used per modality: original images, Laplacian-of-Gaussian (LoG)-filtered images at five sigma levels (1.0–5.0 mm), and a single-level 3D wavelet decomposition using the Coiflet-1 (coif1) kernel, yielding the eight directional sub-bands (LLL,LLH,LHL,LHH,HLL,HLH,HHL,HHH). Intensities were discretized using a fixed bin width prior to texture computation (bin width 5 for the *Z*-score-normalized MRI and 25 HU for CT), applied identically to the original, LoG, and wavelet images. For each image type, we extracted 18 first-order, 14 shape, and 75 texture features (GLCM, GLRLM, GLSZM, GLDM, NGTDM; matrix aggregation detailed in Supplementary Table S3), giving 1316 features per sequence: 3948 for MRI (T1w, T2w Dixon-W, CE T1w Dixon-W) and 1316 for CT (Supplementary Fig. S2).

### 2.4 Machine learning framework

**Fig. 1:**
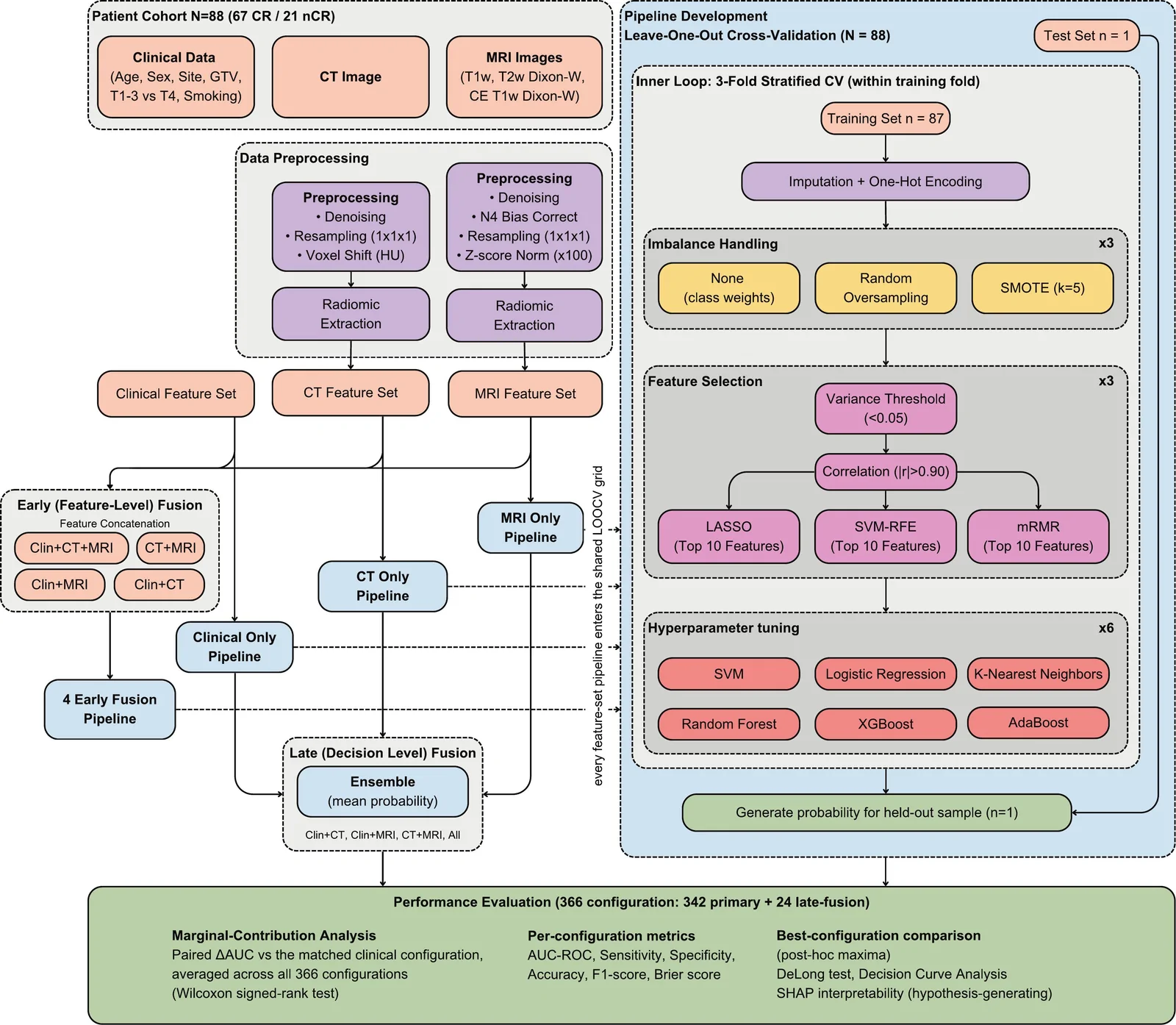
Study workflow illustrating image preprocessing, the Leave-One-Out Cross-Validation (LOOCV) framework, and the multi-stage machine learning pipeline for predicting nCR.

We compared predictive models from three data types (clinical, CT radiomics, MRI radiomics) and their multimodal fusions. To address the limited sample size (*N* = 88), all models were trained using nested cross-validation based on Leave-One-Out Cross-Validation (LOOCV; 88 outer iterations, Fig. 1). The clinical model comprised age, sex, primary tumor site, T category (dichotomized as T1–3 vs T4), smoking status, and gross primary tumor volume. In each iteration, one patient was held out, and all data-dependent steps (imputation, one-hot encoding of the categorical clinical variables [sex, site, smoking], scaling, class-imbalance handling, and feature selection) were fit only on the 87 training cases and applied to the held-out patient, preventing leakage; hyperparameters were tuned by an inner 3-fold stratified grid search.

Three class-imbalance strategies were compared: class-weighted training (baseline), Random Oversampling, and Synthetic Minority Over-sampling Technique (SMOTE) [19]; synthetic or duplicated samples were excluded from all evaluations. Feature reduction proceeded in three steps: variance filtering (threshold <0.05), redundancy pruning (absolute Pearson correlation >0.90, retaining the first feature of each correlated pair), and selection of the top 10 features by LASSO [20], Support Vector Machine methods based on Recursive Feature Elimination (SVM-RFE) [21], and the Minimum Redundancy Maximum Relevance (mRMR) [22]. The selection was limited to a maximum of 10 features for each data type and selection method. This ensured all data types had the same maximum signature size, preventing model complexity from affecting comparisons. Six classifiers were evaluated: Logistic Regression (LogReg), Support Vector Machine (SVM), Random Forest, XGBoost [23], AdaBoost [24], and K-Nearest Neighbors (KNN), yielding 366 model configurations in total. The search space for hyperparameters is detailed in Supplementary Table S4.

### 2.5 Multimodal fusion strategies

Two fusion approaches were tested, each in four combinations (Clinical+CT + MRI, Clinical+CT, Clinical+MRI, CT + MRI). Early fusion concatenated features from all included sources into a single vector before the pipeline, letting the selection algorithms identify markers across sources. Late fusion was a decision-level ensemble: separately optimized models for each modality (same classifier type) whose predicted probabilities were averaged with equal weights (Supplementary Section 3). To investigate feature competition, the features selected in the joint early-fusion space were compared with those from the standalone models, and the proportions retained and displaced were recorded.

### 2.6 Statistical analysis and clinical utility

Baseline characteristics were compared between CR and nCR using Fisher's exact test (categorical) and the independent *t*-test or Mann-Whitney *U* test (continuous) at *p* < 0.05. Discrimination was AUC from aggregated out-of-fold LOOCV predictions; the classification threshold was set by maximizing Youden's J statistic [25], and 95% confidence intervals were obtained from 1000 bootstrap resamples. AUC differences were tested with the DeLong test [26] (*p* < 0.05). Decision curve analysis (DCA) [27] was performed on logistic-recalibrated (Platt scaling [28]) out-of-fold predictions to assess clinical utility.

To quantify each data type's contribution independently of any single, optimistically selected pipeline, we estimated its marginal contribution across all configurations while controlling for imbalance handling, feature selection, and classifier. For each matched combination, we computed the paired difference in AUC between the data type and the clinical baseline, summarized it as the mean paired difference (with 95% CI), and performed a Wilcoxon signed-rank test across all 366 configurations. Because all configurations share the same 88 patients, a patient-level bootstrap (2000 resamples) is also reported (Supplementary Section 4).

For interpretability, a final model was retrained on all 88 patients with the optimal hyperparameters, and SHAP values [29] were computed. Selection stability was assessed by recording how often each feature appeared across all 88 LOOCV folds. Associations between top radiomic features and categorical clinical variables were tested with the Mann-Whitney U or Kruskal-Wallis test. All analyses used Python (v3.12.3) with scikit-learn (v1.7.2), imbalanced-learn (v0.14.0), RadiomiX [30], and the SHAP library (v0.50.0).

## 3 Results

### 3.1 Patient characteristics

The cohort comprised 88 patients with locally advanced HNSCC: 67 (76.1%) responders (CR) and 21 (23.9%) non-responders (nCR). Baseline characteristics are summarized in Table 1. Primary tumor site was significantly associated with treatment outcome (*p* < 0.001). Oral cavity tumors had the highest non-response rate (64.3%), whereas oropharyngeal and laryngeal tumors more often responded. Larger GTVp (median 66.8 vs 20.7 cm^3^, *p* = 0.006) and T4 category (nCR 32.6% vs 14.3% for T1–3; dichotomized *p* = 0.050) were also associated with non-response.

**Table 1:** Baseline demographic and clinical characteristics of the study cohort.

Characteristic	Total (*N* = 88)	CR group (*n* = 67)	nCR group (*n* = 21)	*p*-value
**Age (years)**				0.961
Mean ± SD	61.8 ± 10.1	61.8 ± 9.6	61.7 ± 12.1	

**Gender**				0.470
Male	76 (86.4%)	59 (88.1%)	17 (81.0%)	
Female	12 (13.6%)	8 (11.9%)	4 (19.0%)	

**Primary Site**				< 0.001*
Oral cavity	14 (15.9%)	5 (7.5%)	9 (42.9%)	
Oropharynx	31 (35.2%)	28 (41.8%)	3 (14.3%)	
Hypopharynx	20 (22.7%)	13 (19.4%)	7 (33.3%)	
Larynx	23 (26.1%)	21 (31.3%)	2 (9.5%)	

**Tumor Classification**				0.050^†^
T1	2 (2.3%)	2 (3.0%)	0 (0%)	
T2	7 (8.0%)	6 (9.0%)	1 (4.8%)	
T3	33 (37.5%)	28 (41.8%)	5 (23.8%)	
T4	46 (52.3%)	31 (46.3%)	15 (71.4%)	

**Nodal classification**				0.902
N0	27 (30.7%)	21 (31.3%)	6 (28.6%)	
N1	12 (13.6%)	9 (13.4%)	3 (14.3%)	
N2	29 (33.0%)	23 (34.3%)	6 (28.6%)	
N3	20 (22.7%)	14 (20.9%)	6 (28.6%)	

**Smoking**				0.377
Yes	69 (78.4%)	54 (80.6%)	15 (71.4%)	
No	19 (21.6%)	13 (19.4%)	6 (28.6%)	

**GTVp (cm**^**3**^**)**				0.006
Median (IQR)	31.5 (11.9–73.0)	20.7 (8.6–58.6)	66.8 (31.5–75.9)	

Note: Data are n (%) unless indicated; age is mean ± SD, GTVp volume median (IQR). Tests: *t*-test (age), Mann-Whitney U (GTV volume), Fisher's exact test (categorical). Abbreviations: CR, complete response; nCR, non-complete response; SD, standard deviation; GTVp, gross primary tumor volume; IQR, interquartile range. * *p* < 0.05.

^†^ The T-classification p-value refers to the dichotomized T1–3 versus T4 comparison used in the clinical model (Fisher's exact); the four T categories are shown for completeness.

### 3.2 Marginal contribution of each data type

**Fig. 2:**
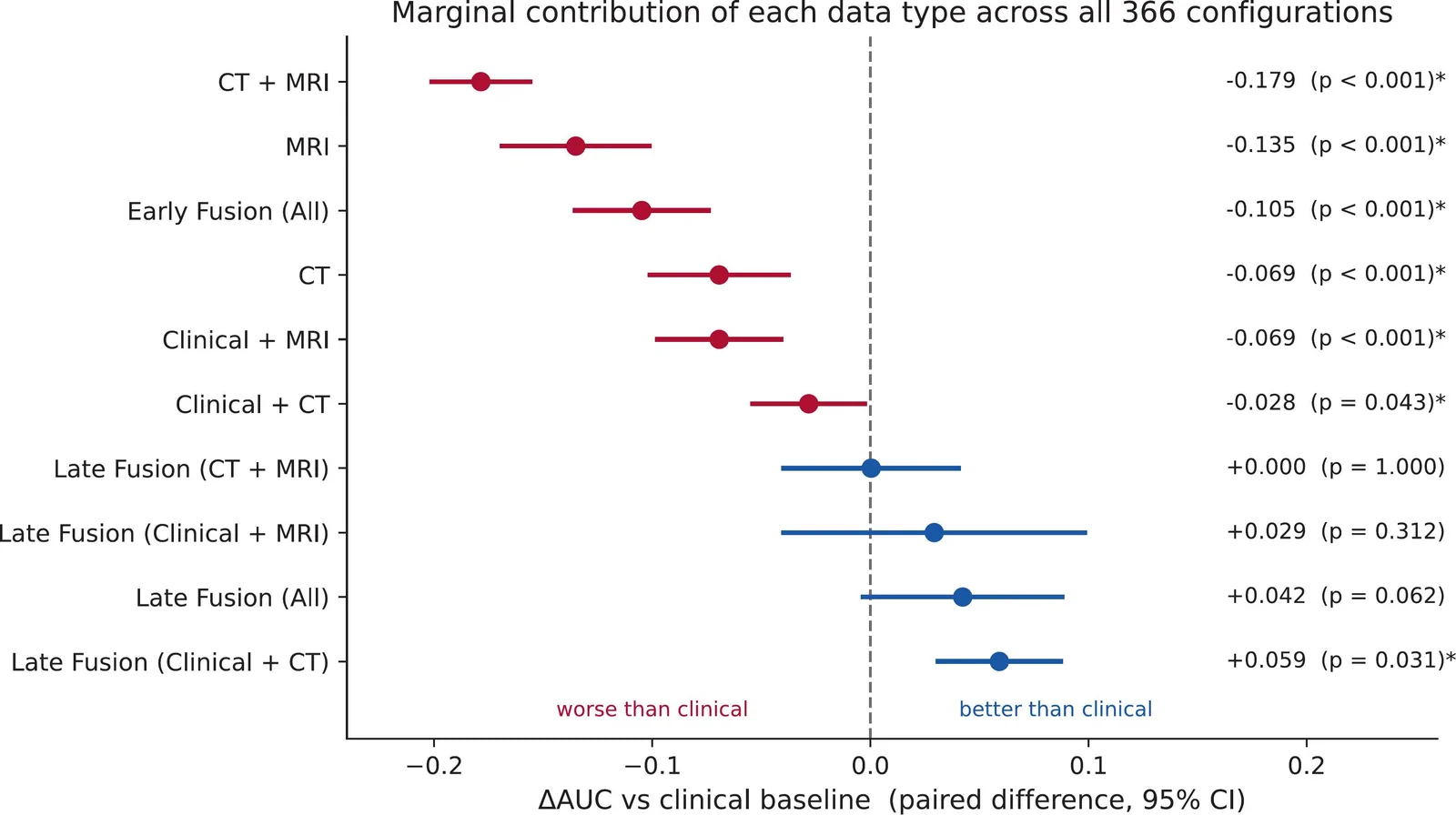
Marginal contribution of each data type relative to the clinical baseline across all 366 configurations. Points are the mean paired ΔAUC vs clinical (blue above, red below the dashed baseline); whiskers are 95% CIs, with Wilcoxon signed-rank *p*-values. (For interpretation of the references to colour in this figure legend, the reader is referred to the web version of this article.)

Each data type's marginal contribution relative to the clinical baseline across all 366 configurations is summarized in Table 2 and Fig. 2 (estimation detail in Supplementary Section 4). No imaging-only approach exceeded the clinical baseline on average. Every single-modality and feature-level (early) fusion model performed below it consistently across configurations. Only decision-level (late) fusion combining the clinical model with imaging was positive, consistently so for Late Fusion (Clinical+CT) alone, whereas late fusion of CT and MRI without clinical data was null. A patient-level bootstrap widened the single-modality and early-fusion intervals approximately three to five times, though every direction was unchanged. Thus, imaging added value only when combined with the clinical model; the single-best-pipeline view is more optimistic and unstable (Section 3.3).

**Table 2:** Marginal contribution of each data type across all 366 configurations.

Feature Set	Mean AUC	Best AUC	ΔAUC vs Clinical	Configuration-level 95% CI	Wilcoxon p	Patient-level bootstrap 95% CI
**Single Modality**						
Clinical	0.683	0.755	–	–	–	
CT	0.614	0.729	−0.069	(−0.101, −0.038)	< 0.001	(−0.172, +0.041)
MRI	0.548	0.910	−0.135	(−0.169, −0.101)	< 0.001	(−0.246, −0.021)

**Early Fusion**						
Early (All)	0.578	0.780	−0.105	(−0.135, −0.074)	< 0.001	(−0.190, −0.008)
Early (CT + MRI)	0.505	0.679	−0.179	(−0.201, −0.156)	< 0.001	(−0.290, −0.069)
Early (Clin+CT)	0.655	0.774	−0.028	(−0.054, −0.003)	0.043	(−0.111, +0.065)
Early (Clin+MRI)	0.614	0.737	−0.069	(−0.098, −0.041)	< 0.001	(−0.155, +0.018)

**Late Fusion**						
Late (All)	0.725	0.792	+0.042	(−0.003, +0.088)	0.062	(−0.033, +0.119)
Late (CT + MRI)	0.683	0.751	0.000	(−0.040, +0.040)	1.000	(−0.109, +0.108)
Late (Clin+CT)	0.742	0.789	+0.059	(+0.031, +0.087)	0.031	(+0.007, +0.118)
Late (Clin+MRI)	0.712	0.822	+0.029	(−0.040, +0.098)	0.312	(−0.031, +0.090)

Note: ΔAUC is the mean paired difference versus the clinical baseline within matched configurations; the Configuration-level 95% CI is the t-interval across configurations, and the p-value is from the Wilcoxon signed-rank test. Single-modality and early fusion rows are matched pairs; late-fusion rows are matched by classifier type (6 pairs). Patient-level bootstrap 95% CI: percentile interval from 2000 patient-level resamples (Supplementary Section 4).

A variance decomposition confirmed these modeling choices as major sources of the spread: after data type, the classifier (η^2^ 7.2%) and feature selector (η^2^ 4.0%) contributed most to AUC variance, and the best combination differed by data type (interaction η^2^ 10.5%; all permutation *p* < 0.02; Supplementary Section 6). Within a data type, the mean difference from clinical ranged widely across pipelines (Supplementary Fig. S3). The balanced grid averages over this spread without bias, but it is the same spread from which a single post-hoc best configuration is drawn, underscoring why one optimized pipeline can overstate imaging's added value.

### 3.3 Best-configuration performance

Following the common practice of reporting a single best-performing model per data type, Table 3 summarizes the best pipeline for each modality and fusion strategy (Youden-optimal threshold; full configurations and secondary metrics in Supplementary Table S7). Even in this optimistic view, the best MRI model (LASSO + SVM) reached an AUC of 0.91 (95% CI: 0.81–0.98) but was not significantly better than the parsimonious clinical model (AUC 0.75, DeLong *p* = 0.058), although it exceeded the best CT model (AUC 0.73, DeLong *p* = 0.017). These are post-hoc maxima without multiplicity correction: the advantage does not survive marginal estimation (Section 3.2), and the gap between the best MRI AUC (0.910) and its across-configuration average (0.548) reflects instability rather than a robust signal. The best early-fusion models did not surpass standalone MRI, reflecting the displacement of each modality's optimized features when pooled (Supplementary Table S5).

**Table 3:** Performance comparison of single-modality and fused models for predicting nCR.

Feature Set	Configuration (Model/ Sampler/ Selector)	AUC (95% CI)	Sensitivity (95% CI)	Specificity (95% CI)	PPV (95% CI)	NPV (95% CI)
**Single Modality**						
Clinical	AdaBoost / SMOTE / None	0.75 (0.62–0.87)	0.76 (0.57–0.93)	0.79 (0.69–0.89)	0.53 (0.36–0.73)	0.91 (0.84–0.98)
CT	XGBoost / None / LASSO	0.73 (0.61–0.84)	0.90 (0.75–1.00)	0.47 (0.34–0.59)	0.35 (0.23–0.48)	0.94 (0.84–1.00)
MRI	SVM / None / LASSO	0.91 (0.80–0.99)	0.90 (0.76–1.00)	0.88 (0.80–0.95)	0.70 (0.52–0.86)	0.97 (0.92–1.00)

**Early Fusion**						
Early (All)	SVM / SMOTE / mRMR	0.78 (0.66–0.88)	0.95 (0.83–1.00)	0.55 (0.43–0.67)	0.40 (0.26–0.55)	0.97 (0.91–1.00)
Early (CT + MRI)	SVM / None / LASSO	0.69 (0.55–0.81)	0.67 (0.47–0.88)	0.67 (0.56–0.78)	0.39 (0.24–0.56)	0.87 (0.78–0.96)
Early (Clin+CT)	LogReg / SMOTE / mRMR	0.77 (0.65–0.88)	0.81 (0.63–0.96)	0.72 (0.61–0.82)	0.47 (0.30–0.64)	0.92 (0.84–0.98)
Early (Clin+MRI)	KNN / SMOTE / LASSO	0.74 (0.62–0.86)	0.76 (0.58–0.94)	0.69 (0.57–0.80)	0.43 (0.28–0.60)	0.90 (0.82–0.98)

**Late Fusion**						
Late (All)	Ensemble (SVM)	0.79 (0.65–0.90)	0.71 (0.50–0.89)	0.81 (0.71–0.89)	0.54 (0.36–0.72)	0.90 (0.82–0.96)
Late (CT + MRI)	Ensemble (SVM)	0.75 (0.62–0.87)	0.86 (0.70–1.00)	0.67 (0.56–0.78)	0.45 (0.29–0.61)	0.94 (0.87–1.00)
Late (Clin+CT)	Ensemble (AdaBoost)	0.79 (0.65–0.90)	0.76 (0.57–0.94)	0.85 (0.77–0.93)	0.62 (0.42–0.80)	0.92 (0.85–0.98)
Late (Clin+MRI)	Ensemble (SVM)	0.82 (0.72–0.91)	0.71 (0.50–0.91)	0.82 (0.72–0.91)	0.56 (0.35–0.73)	0.90 (0.82–0.97)

Note: Best pipeline per group as Model / Sampler / Selector (clinical-only uses no selector); late-fusion rows are decision-level ensembles of the best per-modality models. Abbreviations: AdaBoost: Adaptive Boosting; AUC: Area Under the Receiver Operating Characteristic Curve; CI: Confidence Interval; KNN: K-Nearest Neighbors; LASSO: Least Absolute Shrinkage and Selection Operator; LogReg: Logistic Regression; mRMR: minimum Redundancy Maximum Relevance; NPV: Negative Predictive Value; PPV: Positive Predictive Value; SMOTE: Synthetic Minority Over-sampling Technique; SVM: Support Vector Machine; XGBoost: Extreme Gradient Boosting.

### 3.4 Exploratory characterization of the best-configuration MRI model

Because the best-configuration MRI model did not surpass the clinical baseline in the marginal-contribution analysis (Section 3.2), the clinical-utility and feature analyses below are exploratory and hypothesis-generating. As a best-case illustration of clinical utility, DCA on the recalibrated best-configuration models showed the MRI model with higher net benefit than the clinical baseline and the default “Treat All”/”Treat None” strategies across most of the clinically relevant threshold range (approximately 10% to 65% in Supplementary Fig. S5).

**Fig. 3:**
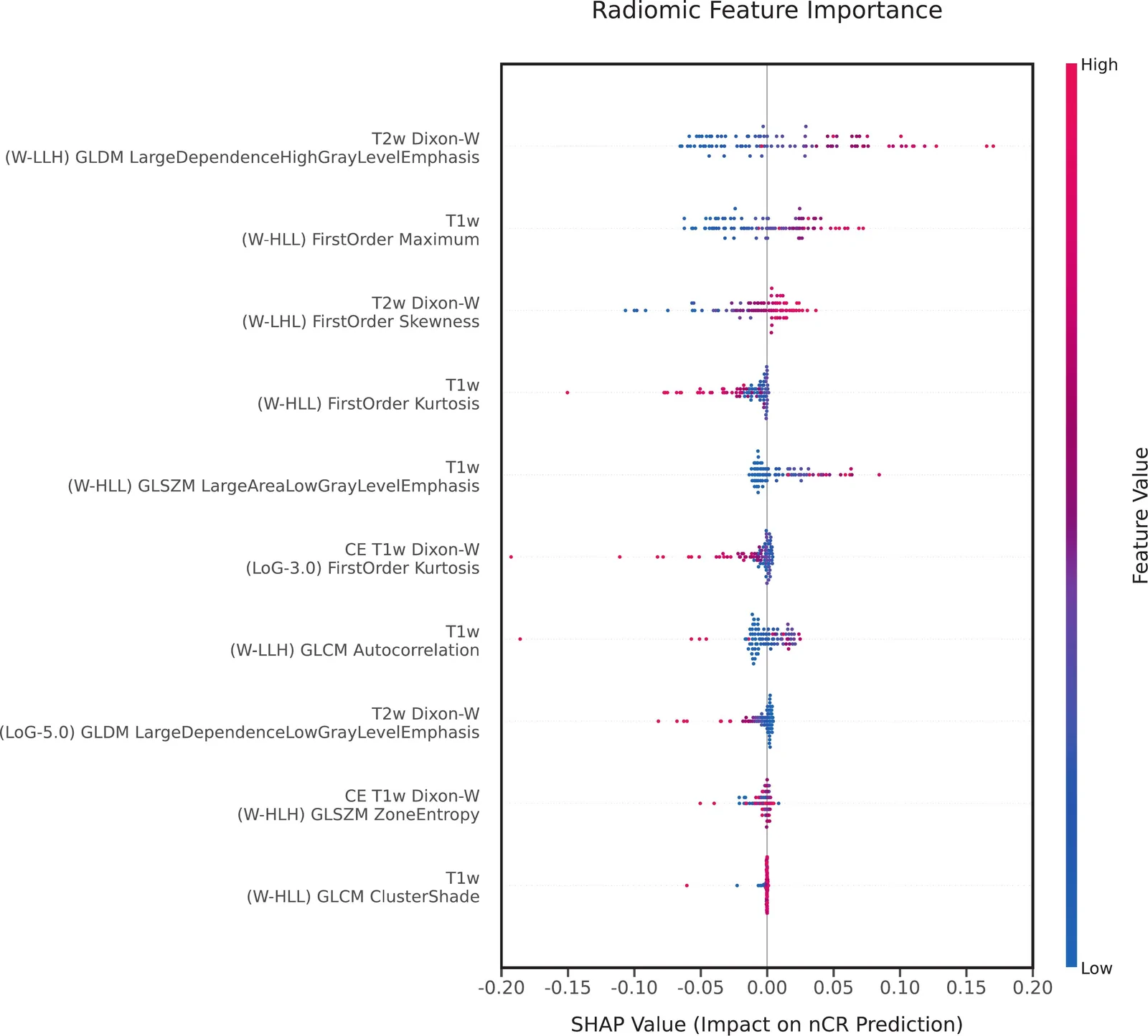
SHAP summary plot illustrating the top 10 features contributing to the prediction of nCR in the final MRI-based global model. Features are ranked by mean absolute SHAP value. Red indicates high feature values, and blue indicates low feature values. A shift to the right (positive SHAP) indicates a higher probability of nCR. (For interpretation of the references to colour in this figure legend, the reader is referred to the web version of this article.)

A global fit of the best MRI pipeline (LASSO+SVM) on all 88 patients yielded a 10-feature signature spanning T1w, T2w Dixon-W, and CE T1w Dixon-W, dominated by wavelet- and LoG-filtered features (Fig. 3). Non-response was associated with higher values of the top feature, T2w Dixon-W wavelet-LLH GLDM Large Dependence High Gray-Level Emphasis (coarse, high-intensity texture), with higher intensity extremes (T1w wavelet-HLL FirstOrder Maximum), and with lower kurtosis (a broader, flatter intensity distribution).

Phenotypic mapping tied this signature to clinical hallmarks: the top feature was associated with primary tumor site and T-stage (both *p* < 0.001), and most top-ranked features with T-stage (7 of 10, *p* < 0.01; Supplementary Table S9). Across the 88 LOOCV folds, all 10 features were selected in over 50% of folds and the top three in over 93% (Supplementary Fig. S6), indicating stable selection within the best-configuration pipeline. This overlap indicates that the signature largely re-encodes information already in the clinical baseline (primary site and T-stage), consistent with its lack of independent marginal value.

## 4 Discussion

In this single-center retrospective cohort of 88 patients with unresectable LA-HNSCC treated with definitive concurrent chemoradiotherapy, we compared clinical, CT-based, and MRI-based machine-learning models for predicting non-complete response at the primary tumor. In a best-configuration comparison, a multi-sequence MRI signature reached the highest discrimination (AUC 0.91) but did not reach statistical significance against a parsimonious clinical model (DeLong *p* = 0.058). When marginal contributions were evaluated across all 366 configurations, neither single-modality nor feature-level fusion models improved upon the clinical baseline on average; improvement was observed only with decision-level fusion of clinical and imaging data. The high single-best MRI AUC therefore reflects favorable configuration selection rather than a stable modality-level advantage over a competitive clinical baseline.

Our baseline data reinforced primary tumor site as a key factor in early treatment outcomes. Oral cavity and hypopharyngeal tumors had higher nCR rates despite comparable treatment intensity, aligning with reports of poorer results with non-surgical management at these sites [31], [32]. Non-responders were more likely to have T4 disease, consistent with lower complete response rates reported for T3-T4 laryngeal and hypopharyngeal cancers [33]. Gross primary tumor volume was markedly larger in non-responders, consistent with the prognostic role in head and neck radiotherapy [34]. These compact clinical markers of early failure help explain why high-dimensional radiomics struggled to improve on a model built from these variables.

Previous HNSCC radiomics studies report that texture signatures can predict response, but with variable performance across modalities [5], [13], [14]. Our findings warrant caution: the high performance of the MRI model was sensitive to the choice of classifier and feature-selection method, which accounted for substantial variability in model performance. This accords with Kaźmierska et al. [13], who found that clinical factors (AUC 0.78) were not improved by adding CT radiomics; in our cohort, CT radiomics likewise did not exceed the clinical baseline, alone or through feature-level fusion. Building on prior HNSCC radiomics works by Corti et al. [14] and Bologna et al. [35], we additionally targeted the short-term nCR endpoint and quantified each data type's marginal contribution across all configurations rather than a single best pipeline.

The fused models clarify when combining data types helps. Early fusion consistently reduced performance relative to the best MRI model. Pooling CT and MRI features led regularized selection to displace features that were optimal in the standalone MRI model, yielding a weaker composite. In contrast, only decision-level fusion that included the clinical model exceeded the clinical baseline, whereas late fusion of CT and MRI alone was null. The benefit therefore arose from combining imaging with clinical information, indicating that the integration strategy and the inclusion of clinical data, not imaging modalities alone, govern whether multimodal information helps in small cohorts [14], [36].

Interpretation of the best-configuration MRI signature is hypothesis-generating. Composed entirely of wavelet- and LoG-filtered features, it captures multi-scale texture detail not readily apparent on conventional visual review; such heterogeneous patterns are consistent with intratumoral heterogeneity, which has been linked to treatment resistance, progression, and recurrence in head and neck cancer and nasopharyngeal carcinoma [37], [38]. Because these features correlated with oral cavity site and advanced T-stage, the signature partly re-encodes the same anatomical and disease-extent information as the clinical baseline, reinforcing why MRI added no stable value beyond it. Its apparent net benefit on decision-curve analysis is likewise a best-case, exploratory estimate that must be confirmed in independent data before it could guide decisions such as dose escalation or intensified systemic therapy.

Several limitations must be acknowledged. The retrospective single-center design and small sample size (*N* = 88, with 21 events) raise a risk of overfitting, which our marginal-contribution analysis was designed to address. Imaging used a single MRI platform, so inter-scanner variability was untested; tumor volumes were manually delineated without blinded review; and response assessment relied on heterogeneous follow-up imaging, predominantly contrast-enhanced CT, which may introduce endpoint misclassification. HPV/p16 status was not routinely tested and could not be incorporated despite being a known determinant of radiation response. PyRadiomics filter implementations are not fully IBSI-compliant. Fusion was limited to feature concatenation and unweighted probability averaging; attention-based or learned-weight approaches might better exploit complementary information.

In this LA-HNSCC cohort, no single-modality or feature-level fusion radiomics model exceeded a parsimonious clinical baseline, and the highest single-configuration result (a multi-sequence MRI model, AUC 0.91) did not reach significance against that baseline. Only decision-level fusion combining imaging with the clinical model surpassed it. Single-best radiomic performance can therefore be optimistic in small cohorts, and prospective, multi-institutional validation is required before radiomics-based risk stratification can guide treatment intensification in LA-HNSCC.

## Declaration of generative AI and AI-assisted technologies in the manuscript preparation process

During the preparation of this work, the authors used Google Gemini (Google LLC, Mountain View, CA, USA) and Claude (Anthropic, PBC, San Francisco, CA, USA) in order to assist with the technical implementation of computational pipelines, including drafting code for scikit-learn workflows, assisting with matplotlib figure formatting, and suggesting syntax corrections for complex loops in the leave-one-out cross-validation framework. After using this tool, the authors reviewed, verified, and edited all generated material as needed and take full responsibility for the content of the published article.

## CRediT authorship contribution statement

**Krittee Chesdachai:** Writing – review & editing, Writing – original draft, Visualization, Methodology, Investigation, Data curation. **Kullathorn Thephamongkhol:** Writing – review & editing, Validation, Supervision, Project administration, Methodology. **Anucha Chaichana:** Writing – review & editing, Visualization, Validation, Software, Project administration, Methodology, Investigation, Formal analysis, Data curation. **Jiraporn Setakornnukul:** Writing – review & editing, Validation, Supervision, Project administration, Methodology, Investigation, Conceptualization.

## Ethics declaration

Written informed consent to take part in the study and to publish the article has been obtained from all participants or their legal representatives. The privacy rights of participants have been observed.

This study was performed in compliance with relevant laws, regulatory frameworks and guidelines where the research took place.

This study was approved by the Siriraj Institutional Review Board, Faculty of Medicine Siriraj, Mahidol University.

(Approval No. MU-MOU COA No. 750/2024)

## Declaration of competing interest

The authors declare that they have no known competing financial interests or personal relationships that could have appeared to influence the work reported in this paper.

## Data Availability

The extraction parameters, analysis code, per-configuration results, and selected-feature lists are openly available at (https://doi.org/10.5281/zenodo.21295298). Patient images and individual-level data cannot be shared publicly owing to ethical restrictions.
