# Supplementary material for: Computed tomography and magnetic resonance imaging radiomics for predicting non-complete response after chemoradiotherapy in unresectable locally advanced head and neck squamous cell carcinoma
Source: Phys Imaging Radiat Oncol. 2026 Sep 5;41:101074. doi: 10.1016/j.phro.2026.101074 (PMC13579444; doi:10.1016/j.phro.2026.101074)
Supplement: Supplementary file 1 [file mmc1.pdf]

Supplementary Material

Computed Tomography and Magnetic Resonance Imaging Radiomics for Predicting Non-Complete Response after Chemoradiotherapy in Unresectable Locally Advanced Head and Neck Squamous Cell Carcinoma

Supplementary Section 1. Study cohort, treatment, and imaging acquisition

Table S1. Treatment and follow-up characteristics of the study cohort (N = 88)

Treatment characteristic	Value
**Radiotherapy dose (EQD2, Gy)**	
- Median	70.7
- Range	58.4-70.7
**Overall treatment time (days)**	
- Median	50
- Range	29-75
**Induction chemotherapy (Yes / No)**	9/79
**Concurrent chemoradiation schedule**	
- Triweekly	50
- Weekly	38
**Chemotherapy regimen**	
- Cisplatin	63
- Carboplatin	18
- Cisplatin switched to carboplatin	7
**Chemotherapy compliance**	
- ≥ 2/3 of planned regimen	78
- < 2/3 of planned regimen	10
**Time from simulation to RT start (days)**	Median 14 (IQR 14-15)
**Time to first follow-up imaging (days)**	Median 96 (IQR 84-122)
**Response assessment imaging modality (CT/MRI)**	70/18
- Non-complete response, CT-assessed	18/70 (25.7%)
- Non-complete response, MRI-assessed	3/18 (16.7%)
- Comparison of non-response rate [a]	OR 0.58 (95% CI 0.15-2.23), p = 0.544
- Time to follow-up imaging, CT-assessed (days)	Median 96 (IQR 85-120)
- Time to follow-up imaging, MRI-assessed (days)	Median 95.5 (IQR 83-126)
- Comparison of imaging interval [b]	p = 0.905
**CT contrast agent**	Iopromide (Ultravist, 370 mgI/mL)
- Dose	50 mL (<50 kg) / 75 mL (≥50 kg), IV, 1-2 mL/s
- Scan delay	60-80 s
**MRI contrast agent**	Gadobutrol (Gadovist, 1.0 mmol/mL)
- Dose	0.1 mL/kg, IV, 1-2 mL/s
- Post-contrast delay	60-80 s
**Median follow-up (months)**	18
**Locoregional recurrence, n**	36
**Death, n**	43

[a] Fisher's exact test; odds ratio for non-complete response in MRI-assessed relative to CT-assessed patients. [b] Mann-Whitney U test. Baseline characteristics of the two assessment groups are compared in Supplementary Section 1. Abbreviations: CI: confidence interval; EQD2: equivalent dose in 2-Gy fractions; IQR: interquartile range; IV: intravenous; n: number of patients; RT: radiotherapy; OR: odds ratio.

Supplementary Table S2. MRI acquisition parameters and sequence specifications

This table summarizes the acquisition parameters for the three MRI sequences utilized in this study: pre-contrast T1-weighted Turbo Spin Echo (T1w), T2-weighted Turbo Spin Echo Modified Dixon with Water-only Reconstruction (T2w Dixon-W), and post-contrast T1-weighted Turbo Spin Echo Modified Dixon with Water-only Reconstruction (CE T1w Dixon-W). All scans were performed on a Philips Ingenia 3 T MRI scanner.

Table S2. MRI acquisition parameters and sequence specifications.

MRI Sequence	TR/TE (ms)	FOV (mm^3)	Matrix	Slice thickness (mm)	Slice gap (mm)	Flip angle (degree)
T1w	450/33	270x239x180	244x216	1.2	0	80
T2w Dixon-W	5616/114	270x237x180	272x159	2.0	0	90
CE T1w Dixon-W	621/14	270x237x180	272x209	2.0	0	90

Abbreviations: CE: contrast-enhanced; FOV: field of view; TE: echo time; TR: repetition time.

Supplementary Figure S1. Segmentation example

Representative axial slice showing manual segmentation of the gross tumor volume of the primary tumor (GTVp) as a region of interest (ROI) on CE-CT, T1w, T2w Dixon-W, and CE T1w Dixon-W, respectively.

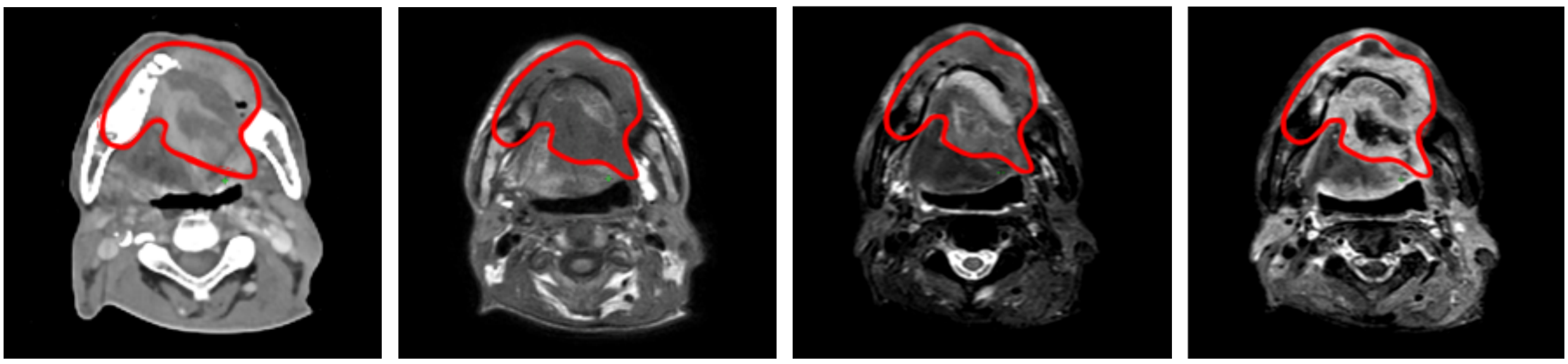

Figure S1. Segmentation example.

Supplementary Section 2. Radiomic feature extraction and model-training configuration

Complete extraction configuration for each modality, provided to enable independent reproduction and external validation.

Table S3. Radiomic feature-extraction settings (PyRadiomics v3.1.1).

Setting	CT	MRI (T1W, T2FS, T1GD)
Intensity units / normalization	Hounsfield Units; no normalization	Whole-image Z-score, scaled ×100 (mean/SD from all image voxels)
Voxel array shift	1024	0
Resampling	1 × 1 × 1 mm^3, B-spline	1 × 1 × 1 mm^3, B-spline
Denoising / bias correction	3D recursive Gaussian (σ = 0.5 mm)	3D recursive Gaussian (σ = 0.5 mm) + N4ITK bias-field correction
Discretization	Fixed bin width = 25 HU	Fixed bin width = 5
Discretization of filtered maps	Same fixed bin width applied to Original, LoG, and Wavelet images	Same fixed bin width applied to Original, LoG, and Wavelet images
LoG filter (σ)	1.0, 2.0, 3.0, 4.0, 5.0 mm	1.0, 2.0, 3.0, 4.0, 5.0 mm
Wavelet kernel	Coiflet-1 (coif1), single-level 3D (8 sub-bands)	Coiflet-1 (coif1), single-level 3D (8 sub-bands)
GLCM	Symmetrical; feature computed per direction and averaged over 13 3D directions	Symmetrical; feature computed per direction and averaged over 13 3D directions
GLRLM	Feature computed per direction and averaged over 13 3D directions	Feature computed per direction and averaged over 13 3D directions
GLSZM / GLDM / NGTDM	Single rotation-invariant matrix	Single rotation-invariant matrix
Feature classes	firstorder, shape, GLCM, GLRLM, GLSZM, GLDM, NGTDM	firstorder, shape, GLCM, GLRLM, GLSZM, GLDM, NGTDM

Supplementary Figure S2. Radiomic feature-extraction pipeline

Schematic of the high-dimensional feature-extraction pipeline. Features were extracted from pre-treatment CT and multi-parametric MRI (T1w, T2w Dixon-W, and CE T1w Dixon-W) with PyRadiomics, applying Wavelet and Laplacian of Gaussian (LoG) filters across the shape, first-order, and texture classes for the primary gross tumor volume (GTVp). Each image type yields 18 first-order and 75 texture features, applied to the original image, five LoG-filtered images, and eight Wavelet sub-bands, plus 14 shape features from the segmentation mask; this gives 1,316 features per sequence and 5,264 per patient across CT and the three MRI sequences.

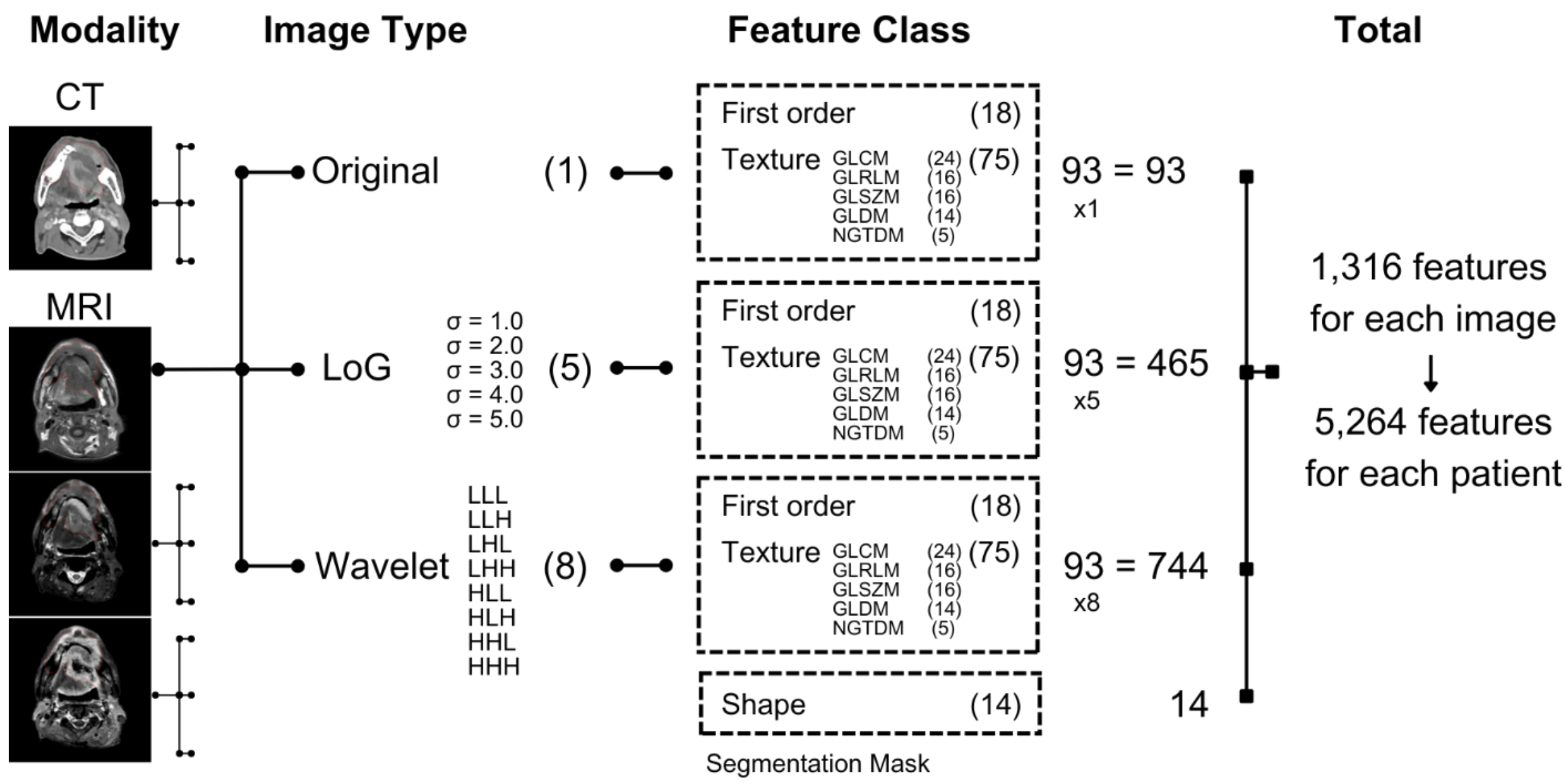

Figure S2. Radiomic feature-extraction pipeline.

Supplementary Table S4. Grid search hyperparameter space for the six machine-learning algorithms

Summary of the hyperparameter optimization space and regularization strategies for the six machine learning algorithms. This table details the specific grid values and architectural constraints used during the inner cross-validation phase to ensure optimal model tuning and prevent overfitting. The 366 configurations comprise 342 primary pipelines (6 radiomic-containing feature sets × 3 imbalance strategies × 3 feature-selection methods × 6 classifiers = 324, plus the Clinical-only set, which bypassed feature selection: 1 × 3 × 1 × 6 = 18) and 24 decision-level late-fusion ensembles.

Table S4. Grid search hyperparameter space for the six machine-learning algorithms

Algorithm	Hyperparameter	Grid Values	Regularization
Logistic Regression	C (Regularization)	[0.1, 1, 10]	L2 penalty
Support Vector Machine	C (Regularization)	[0.1, 1, 10]	L2 penalty
	Kernel	[RBF, Linear]	
Random Forest	Number of Trees	[100]	Ensemble Pruning
	Maximum Depth	[5, 10]	
XGBoost	Number of Trees	[100]	Gradient Shrinkage
	Maximum Depth	[3, 5]	
AdaBoost	Number of Estimators	[50, 100]	Sequential Weighting
	Learning Rate	[0.1, 1.0]	
K-Nearest Neighbors	Number of Neighbors	[3, 5, 7]	Distance Metric

Supplementary Section 3. Multimodal fusion strategies and feature competition

We evaluated two integration strategies to leverage complementary information from Clinical, CT, and MRI data.

1. **Early Fusion (Feature-Level Integration)**

In this approach, available features from multiple data sources were concatenated into a single high-dimensional vector before entering the machine learning pipeline. We evaluated four distinct early fusion combinations: (1) Clinical + CT + MRI, (2) Clinical + CT, (3) Clinical + MRI, and (4) CT + MRI. This joint feature space allowed the selection algorithms to identify the globally optimal set of markers across the combined data sources, potentially capturing synergistic interactions between clinical stages and multimodal radiomic textures.

2. **Late Fusion (Decision-Level Integration)**

Late Fusion was implemented as a decision-level ensemble. Similar to the early fusion approach, we evaluated four distinct late fusion configurations: (1) Clinical + CT + MRI, (2) Clinical + CT, (3) Clinical + MRI, and (4) CT + MRI. For each ensemble member within a given configuration, independent models were developed for each modality (Clinical, CT, and MRI). While the classifier type was consistent across ensemble members (e.g., all models were SVMs), the feature selection methods and hyperparameters were optimized independently for each modality based on their individual LOOCV performance. The final multimodal prediction was then calculated as the unweighted arithmetic mean of the probabilities generated by these modality-specific models:

$$P_{final} = \frac{1}{M}\sum_{i=1}^{M} P_i \qquad \text{Equation 1}$$

Where P_{final} represents the combined multimodal probability of nCR, M denotes the number of modalities included in the specific fusion configuration (2 or 3), and P_i represents the probability output from the i-th modality-specific model. This approach ensured that the ensemble used the same modeling paradigm across data sources while allowing modality-specific tuning to capture the distinctive features of each modality.

Supplementary Table S5. Analysis of feature retention and displacement during early fusion (CT+MRI).

Quantitative analysis of feature retention and displacement during multi-modal integration. This table tracks the survival rate of standalone CT and MRI signatures when pooled into a joint early fusion feature space, illustrating the technical basis for the performance dilution observed in integrated models.

Table S5. Analysis of feature retention and displacement during early fusion (CT+MRI).

Modality	Features in Early Fusion (n = 10)	Features Displaced (%)	Retention of Standalone Top 10
CT Radiomics	3 (30%)	80%	2 / 10
MRI Radiomics	7 (70%)	70%	3 / 10

Supplementary Section 4. Marginal-contribution analysis across all configurations

The paired difference in AUC between each data type and the clinical baseline was formed within matched pipeline factors. For the single-modality and early-fusion sets, the target was averaged over the three feature-selection methods so that each clinical anchor (one per imbalance strategy × learner) contributes once, giving 18 matched pairs; the decision-level late-fusion sets, which have no feature-selection factor, were matched by learner (6 pairs). The clinical model includes no feature-selection step and is therefore matched only by the imbalance strategy and the classifier. Each data type's marginal effect (Table 2) is the mean of these paired differences, with a t-based 95% confidence interval and a Wilcoxon signed-rank test against zero. This estimates the average, configuration-independent contribution of each data type rather than its single best-case performance.

All 366 configurations were fit on the same 88 patients and the same leave-one-out partition, so the interval above describes variability across analytic choices with the patient sample held fixed rather than patient sampling. The configurations are also correlated with one another (median pairwise correlation of out-of-fold predictions 0.38, and 0.39 to 0.67 within a single data type), the same dependence that motivates the permutation test used in Supplementary Section 6. We therefore resampled the 88 patients with replacement 2,000 times, recomputing each configuration's AUC from its stored out-of-fold predictions and re-deriving the matched mean paired difference against the clinical baseline (Supplementary Table S6). Intervals are the 2.5th and 97.5th percentiles, and resamples missing an outcome class were redrawn. Point estimates are unchanged by construction. This resamples patients, unlike the model-level bootstrap behind Supplementary Table S7, which resamples one fitted model's predictions. For the single-modality and early-fusion contrasts, the intervals are three to five times wider, and three that had excluded zero no longer do so; every contrast retains its direction, and both principal findings hold. Because the fitted models are held fixed, these intervals are a lower bound on total uncertainty.

The complete per-configuration performance table underlying Tables 2 and 3 (the main manuscript), all 366 configurations with AUC, accuracy, sensitivity, specificity, and Brier score (Youden-optimal threshold), is provided in Supplementary Data S1 (https://doi.org/10.5281/zenodo.21295298).

Table S6. Patient-level cluster bootstrap of the marginal contribution of each data type (2,000 resamples of N = 88).

Data type	Matched pairs	ΔAUC vs Clinical	Bootstrap SD	Bootstrap 95% CI	Bootstrap p
Early (CT+MRI)	18	−0.179	0.056	(−0.290, −0.069)	0.004
MRI	18	−0.135	0.058	(−0.246, −0.021)	0.022
Early (All)	18	−0.105	0.046	(−0.190, −0.008)	0.033
CT	18	−0.069	0.054	(−0.172, +0.041)	0.182
Early (Clin+MRI)	18	−0.069	0.043	(−0.155, +0.018)	0.094
Early (Clin+CT)	18	−0.028	0.044	(−0.111, +0.065)	0.498
Late (CT+MRI)	6	+0.000	0.055	(−0.109, +0.108)	0.966
Late (Clin+MRI)	6	+0.029	0.030	(−0.031, +0.090)	0.347
Late (All)	6	+0.042	0.038	(−0.033, +0.119)	0.259
Late (Clin+CT)	6	+0.059	0.028	(+0.007, +0.118)	0.023

Supplementary Figure S3. Marginal contribution over the clinical model by pipeline component

Mean difference in AUC relative to the matched clinical configuration (paired within imbalance strategy and classifier, then averaged over imbalance strategies) for each classifier-by-feature-selector combination, shown separately for each data set. Red indicates performance below the clinical baseline and blue above. Within a single data set, the mean paired difference ranged widely (for example, +0.037 to −0.257 for CT), indicating that the estimated marginal contribution is materially dependent on pipeline construction. Because the grid is balanced, this heterogeneity does not bias the configuration-averaged means reported in Table 2 of the main manuscript, but it is the same spread from which a single post-hoc best configuration is drawn.

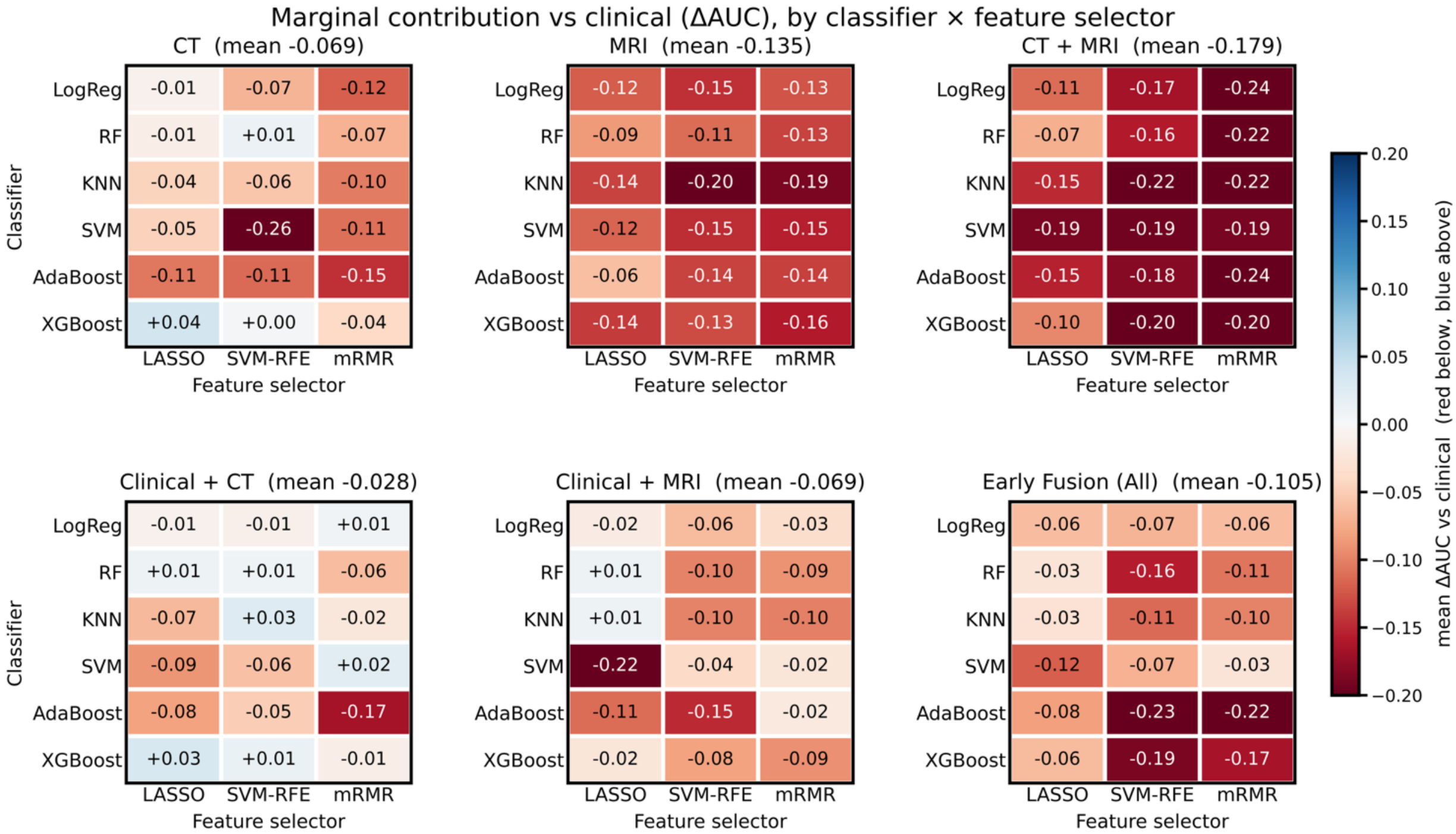

Figure S3. Marginal contribution over the clinical model by pipeline component

Supplementary Section 5. Best-configuration models: technical details and secondary metrics

This table details the technical configurations: classification model, data sampling methods (sampler), and feature selection algorithms of the best-performing models in each category, as summarized in Table 3 of the main manuscript. These configurations represent the optimal pipeline from an exhaustive search of 366 experimental combinations. All metrics, including 95% Confidence Intervals (CIs), were calculated using Leave-One-Out Cross-Validation (LOOCV) to estimate robust performance given the cohort size.

Table S7. Technical Configuration and Secondary Metrics for Best-Performing Models.

Feature Set	Model	Sampler	Selector	AUC (95% CI)	Opt. Thresh	Accuracy (95% CI)	Sensitivity (95% CI)	Specificity (95% CI)	PPV (95% CI)	NPV (95% CI)	F1-Score (95% CI)
**Single Modality**											
Clinical	AdaBoost	SMOTE	None	0.75 (0.62-0.87)	0.49	0.78 (0.69-0.86)	0.76 (0.57-0.93)	0.79 (0.69-0.89)	0.53 (0.36-0.73)	0.91 (0.84-0.98)	0.63 (0.46-0.77)
CT	XGBoost	None	LASSO	0.73 (0.62-0.83)	0.02	0.57 (0.47-0.68)	0.90 (0.78-1.00)	0.46 (0.33-0.59)	0.35 (0.23-0.47)	0.94 (0.85-1.00)	0.50 (0.36-0.63)
MRI	SVM	None	LASSO	0.91 (0.81-0.98)	0.24	0.89 (0.82-0.94)	0.90 (0.76-1.00)	0.88 (0.79-0.95)	0.70 (0.52-0.88)	0.97 (0.92-1.00)	0.79 (0.65-0.91)
**Early Fusion**											
Early (All)	SVM	SMOTE	mRMR	0.78 (0.66-0.88)	0.18	0.65 (0.55-0.75)	0.95 (0.83-1.00)	0.55 (0.43-0.67)	0.40 (0.26-0.55)	0.97 (0.91-1.00)	0.56 (0.41-0.70)
Early (CT+MRI)	SVM	None	LASSO	0.68 (0.54-0.80)	0.25	0.67 (0.57-0.76)	0.67 (0.45-0.87)	0.67 (0.56-0.77)	0.39 (0.23-0.55)	0.87 (0.76-0.96)	0.49 (0.33-0.64)
Early (Clin+CT)	LogReg	SMOTE	mRMR	0.77 (0.65-0.88)	0.40	0.74 (0.65-0.83)	0.81 (0.63-0.96)	0.72 (0.61-0.82)	0.47 (0.30-0.64)	0.92 (0.84-0.98)	0.60 (0.43-0.74)
Early (Clin+MRI)	KNN	SMOTE	LASSO	0.74 (0.62-0.86)	0.57	0.70 (0.60-0.81)	0.76 (0.58-0.94)	0.69 (0.57-0.80)	0.43 (0.28-0.60)	0.90 (0.82-0.98)	0.55 (0.39-0.70)
**Late Fusion**											

Feature Set	Model	Sampler	Selector	AUC (95% CI)	Opt. Thresh	Accuracy (95% CI)	Sensitivity (95% CI)	Specificity (95% CI)	PPV (95% CI)	NPV (95% CI)	F1-Score (95% CI)
Late (All)	Ensemble (SVM)	Ensemble	Ensemble	0.79 (0.65-0.90)	0.40	0.78 (0.70-0.88)	0.71 (0.50-0.89)	0.81 (0.71-0.89)	0.54 (0.36-0.72)	0.90 (0.82-0.96)	0.61 (0.43-0.76)
Late (CT+MRI)	Ensemble (SVM)	Ensemble	Ensemble	0.75 (0.63-0.86)	0.32	0.72 (0.62-0.81)	0.86 (0.70-1.00)	0.67 (0.56-0.78)	0.45 (0.30-0.60)	0.94 (0.86-1.00)	0.59 (0.43-0.72)
Late (Clin+CT)	Ensemble (AdaBoost)	Ensemble	Ensemble	0.79 (0.65-0.90)	0.48	0.83 (0.75-0.91)	0.76 (0.57-0.94)	0.85 (0.77-0.93)	0.62 (0.42-0.80)	0.92 (0.85-0.98)	0.68 (0.50-0.82)
Late (Clin+MRI)	Ensemble (SVM)	Ensemble	Ensemble	0.82 (0.72-0.91)	0.39	0.80 (0.70-0.88)	0.71 (0.50-0.91)	0.82 (0.72-0.91)	0.56 (0.35-0.73)	0.90 (0.82-0.97)	0.62 (0.44-0.78)

Note: The best-performing overall model (MRI Only) is highlighted in bold. Accuracy and F1-score represent the performance on the positive class (nCR) at the optimal threshold (Opt. Thresh) determined via Youden's J statistic. Abbreviations: AdaBoost: Adaptive Boosting; AUC: Area Under the Receiver Operating Characteristic Curve; Clin: Clinical; CT: Computed Tomography; KNN: K-Nearest Neighbors; LASSO: Least Absolute Shrinkage and Selection Operator; LogReg: Logistic Regression; MRI: Magnetic Resonance Imaging; mRMR: Minimum Redundancy Maximum Relevance; nCR: Non-Complete Response; NPV: Negative Predictive Value; PPV: Positive Predictive Value; SMOTE: Synthetic Minority Over-sampling Technique; SVM: Support Vector Machine; XGBoost: Extreme Gradient Boosting.

Supplementary Section 6. Pipeline-component variance decomposition

To quantify the variation in AUC reflected in modeling choices rather than imaging information, the cross-validated AUC was decomposed across the six fully crossed data sets (CT, MRI, CT+MRI, Clinical+CT, Clinical+MRI, and early fusion), each evaluated over the complete factorial grid of three imbalance-handling strategies, three feature selectors, and six classifiers (324 configurations). A fixed-effects analysis of variance was fitted with the data set, imbalance strategy, feature selector, and classifier as main effects and the two data-set-by-component terms (data set x selector, data set x classifier) as a single interaction block. Because each configuration contributes a single LOOCV AUC and these estimates share cross-validation folds and are therefore not independent, significance was assessed using a Freedman-Lane residual permutation test (2000 permutations); the interaction block was tested against the main-effects model to respect marginality. For each term, η^2 (share of total variance) and the bias-adjusted ω^2 are reported (Supplementary Table S8).

The feature selector, which has no counterpart in the clinical model and therefore does not cancel in the paired comparison, shifted the marginal estimate systematically: pooled over classifiers, LASSO gave the least negative and mRMR the most negative contribution for both CT (-0.030 versus -0.098) and MRI (-0.110 versus -0.150). This indicates that the apparent added value of radiomics depends materially on pipeline construction, and that reporting a single optimized configuration mixes imaging information with the choice of selector and classifier.

Proportion of total AUC variance attributable to each source, computed across the 324 fully crossed configurations. η^2 is the raw share of variance and ω^2 the bias-adjusted estimate; p-values are from a Freedman-Lane residual permutation test (2000 permutations). After the data type itself, the classifier and feature selector were the largest identifiable sources of performance variance, whereas the imbalance-handling strategy contributed little. The data type x component interaction (η^2 = 10.5%) indicates that the best-performing component combination differed across data types.

Table S8. Variance decomposition of cross-validated AUC across pipeline choices.

Source of variance	η^2 (%)	ω^2 (%)	Permutation p
Data type (modality)	26.6	25.7	< 0.001
Classifier	7.2	6.2	< 0.001
Feature selector	4.0	3.6	< 0.001
Imbalance-handling strategy	1.6	1.2	0.019
Data type x component interaction	10.5	4.1	0.014
Residual (unexplained)	50.1	n/a	n/a

Supplementary Figure S4. Pipeline component choice as a source of AUC variance

Distribution of cross-validated AUC by feature selector, classifier, and imbalance-handling strategy across the 324 fully crossed configurations. Each point is one configuration; boxes show the median and interquartile range, and the dotted line marks chance (AUC 0.5). The percentage in each panel title is the

share of total AUC variance explained by that component (Supplementary Table S7). Mean AUC ranged from 0.55 (AdaBoost) to 0.63 (logistic regression) across classifiers and from 0.57 (mRMR) to 0.61 (LASSO) across selectors, whereas the imbalance-handling strategy had little effect.

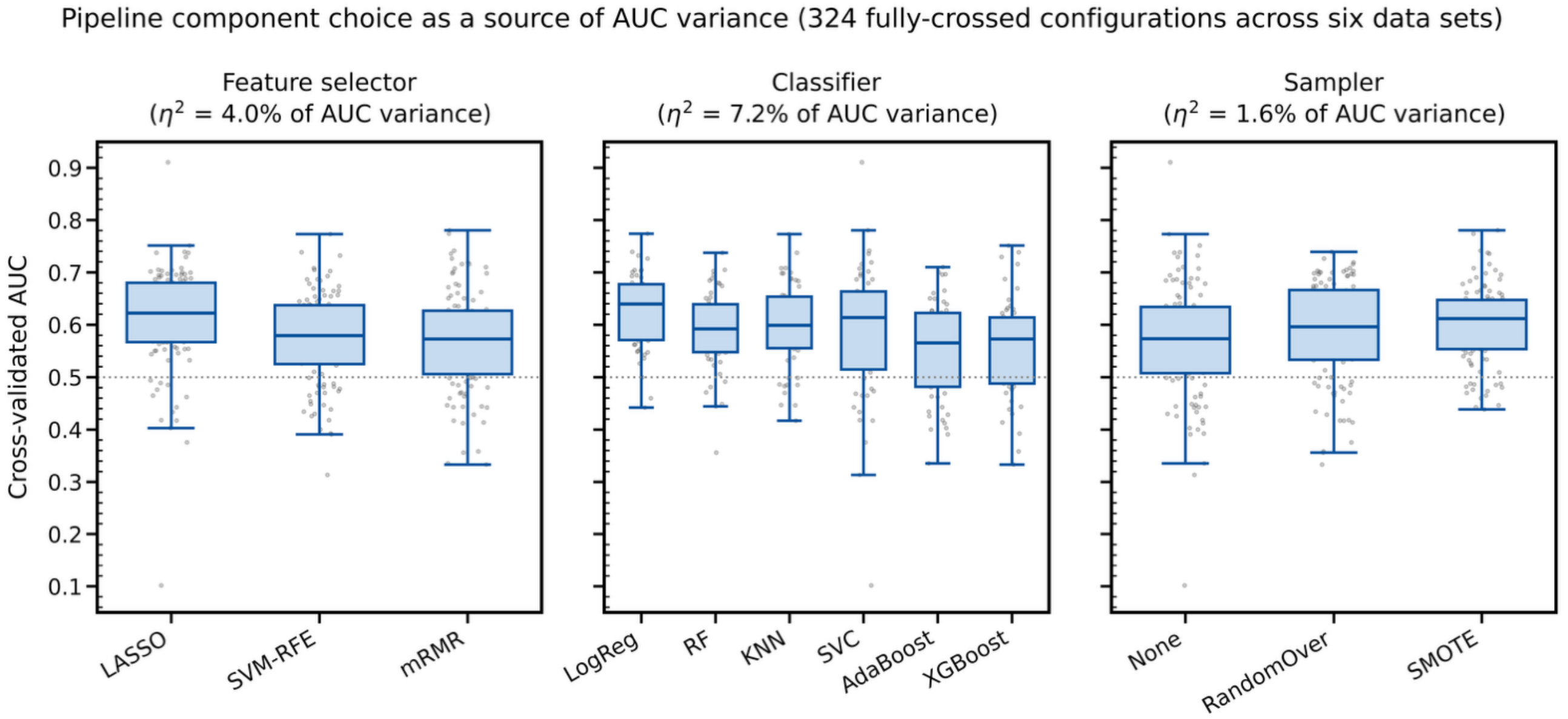

Figure S4. Pipeline component choice as a source of AUC variance.

Supplementary Section 7. Exploratory characterization of the best-configuration MRI model

The best-configuration MRI model did not surpass the clinical baseline in the marginal-contribution analysis; the clinical-utility and feature analyses reported below are therefore exploratory and hypothesis-generating.

Supplementary Figure S5. Decision curve analysis

Net benefit of the recalibrated best-configuration MRI-radiomics model (red) and the clinical baseline (blue) across threshold probabilities, compared with the default "Treat All" and "Treat None" strategies. Curves are logistic-recalibrated (Platt scaling) out-of-fold predictions and are for exploratory purposes. Within this best-case view, the MRI model showed higher net benefit than the clinical baseline across the clinically relevant mid-threshold range.

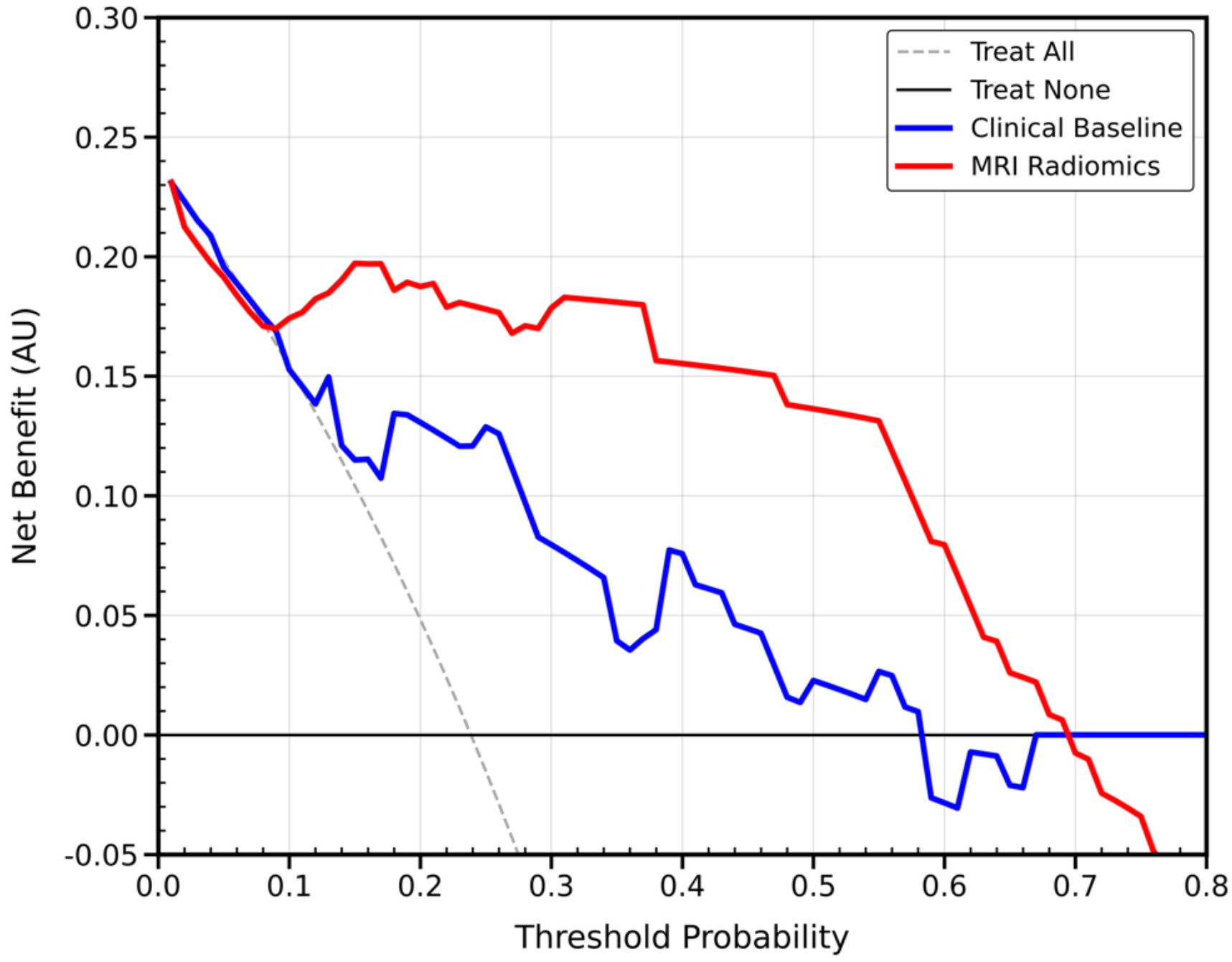

Figure S5. Decision curve analysis.

Supplementary Table S9. Phenotypic correlation analysis

Statistical associations between the top 10 radiomic features of the best-configuration MRI-only signature and baseline categorical clinical variables. P-values indicate the strength of association between each radiomic texture and the clinical hallmark.

Table S9. Phenotypic correlation analysis.

Top 10 MRI Radiomic Features	Sex (p)	Primary Site (p)	T-stage, T1-3 vs T4 (p)	N-stage (p)	Smoking (p)
T2w Dixon-W W-LLH GLDM LargeDepHighGrayEmphasis	0.191	< 0.001*	< 0.001*	0.220	0.516
T1w W-HLL GLSZM LargeAreaLowGrayLevelEmphasis	0.047*	0.640	< 0.001*	0.120	0.191
T2w Dixon-W LoG-5.0 GLDM LargeDepLowGrayLevelEmphasis	0.500	0.060	0.003*	0.186	0.394
T1w W-HLL FirstOrder Maximum	0.515	< 0.001*	< 0.001*	0.185	0.700
T2w Dixon-W W-LHL FirstOrder Skewness	0.023*	0.356	0.263	0.140	0.423
T1w W-HLL FirstOrder Kurtosis	0.150	< 0.001*	< 0.001*	0.165	0.800
CE T1w Dixon-W LoG-3.0 FirstOrder Kurtosis	0.340	0.020*	< 0.001*	0.016*	0.453
T1w W-LLH GLCM Autocorrelation	0.256	< 0.001*	< 0.001*	0.092	0.960
CE T1w Dixon-W W-HLH GLSZM ZoneEntropy	0.005*	0.015*	0.289	0.013*	0.016*
T1w W-HLL GLCM ClusterShade	0.299	0.124	0.467	0.224	0.769

**Statistically significant ($p < 0.05$). Associations tested with the Mann-Whitney U test (Sex, Smoking, and T-stage dichotomized as T1–3 vs T4 to match the clinical model) or the Kruskal-Wallis test (Primary Site, N-stage).*

Most top-ranked features were significantly associated with primary tumor site (6 of 10, $p < 0.05$) and with T-stage (7 of 10, $p < 0.01$), with the top feature reaching $p < 0.001$ for both, indicating that the signature partly re-encodes anatomical subsite and macroscopic disease extent already captured by the clinical baseline. This shared information helps explain why MRI did not add stable value beyond clinical variables in the marginal-contribution analysis.

Supplementary Figure S6. Feature stability analysis

Stability analysis of the final MRI-radiomic signature. This figure was generated by tracking the selection frequency of each top-ranked feature across the 88 independent iterations of the Leave-One-Out Cross-Validation (LOOCV) framework. In each fold, a complete feature selection process (LASSO) was performed on a unique training subset (N = 87).

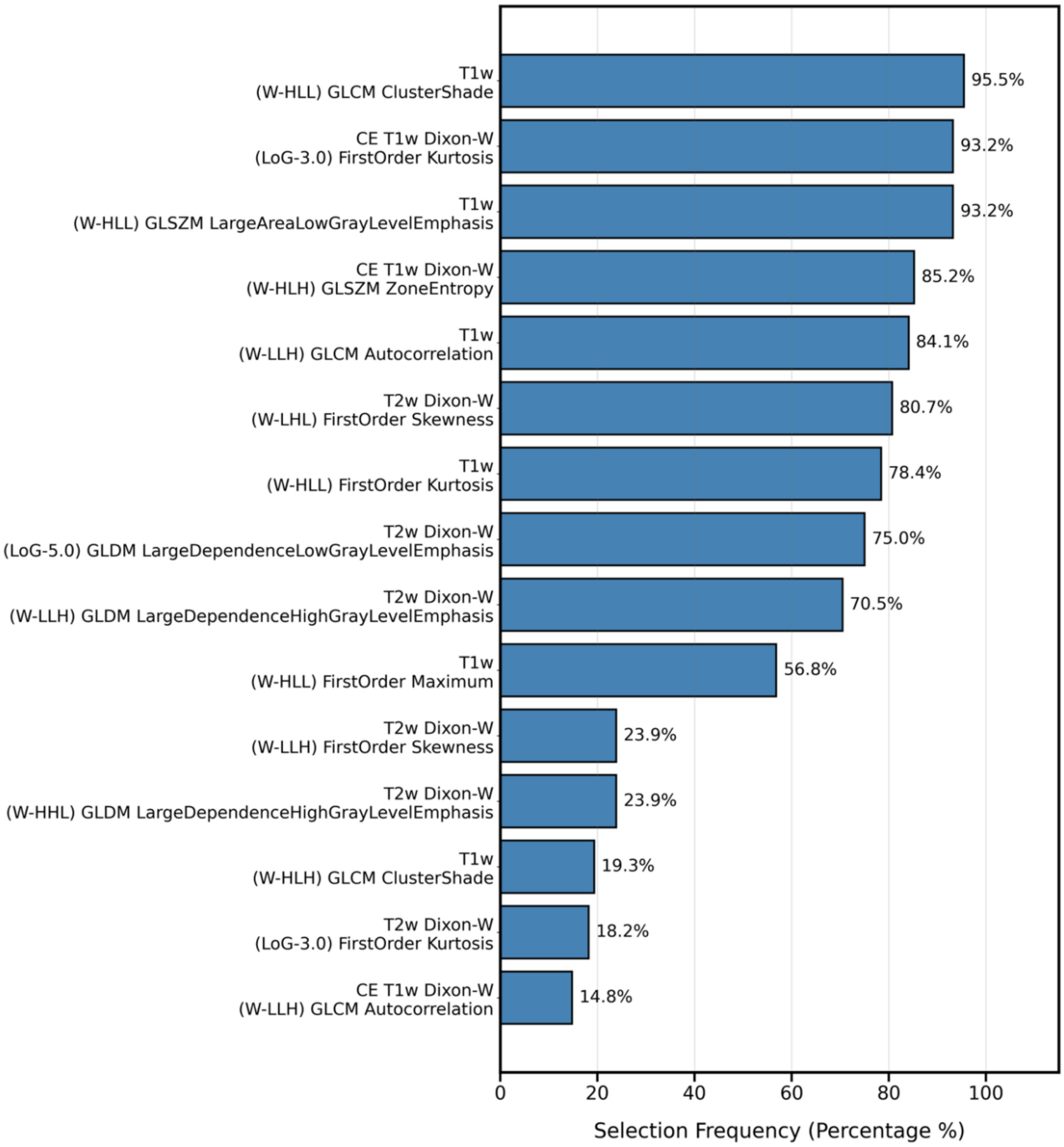

Figure S6. Feature stability analysis.
